# Understanding transitions in exploration profiles of students opting for higher education

**DOI:** 10.3389/fpsyg.2023.1085718

**Published:** 2023-02-09

**Authors:** Lien Demulder, Karine Verschueren, Vincent Donche

**Affiliations:** ^1^School Psychology and Development in Context, Faculty of Psychology and Educational Sciences, KU Leuven, Leuven, Belgium; ^2^Department of Training and Education Sciences, Faculty of Social Sciences, University of Antwerp, Antwerp, Belgium

**Keywords:** study choice, transition to higher education, latent profile analysis, latent transition analysis, person-centered

## Abstract

**Introduction:**

Since previous research on educational career exploration has mainly been cross-sectional and therefore has been unsuccessful in explaining how this process can change during the final year in secondary education before students make the transition to higher education, this study aimed to examine changes over time in the exploration process. A person-centered research perspective was taken to further deepen the understanding of how different exploration tasks jointly combine into meaningful profiles. In this way, this study tried to gain more insight into why some students go through this process successfully and others do not. Four goals guided this study: identifying exploration profiles of students in Fall and Spring of the final year in secondary school based on four decisional tasks (orientation, self-, broad and in-depth exploration), investigating transitions between exploration profiles across these two timepoints, and examining the role which different antecedents (i.e., academic self-efficacy, academic self-concept, motivation, test anxiety, gender, educational track, socio-economic status) play in explaining both profile membership and transitions between profiles.

**Methods:**

Using self-report questionnaires to measure the exploration tasks and the antecedents in final year students, two cross-sectional samples collected in Fall (*n* = 9,567) and Spring (*n* = 7,254), and one longitudinal sample (*n* = 672) were examined.

**Results:**

Latent profile analyses identified three exploration profiles at both timepoints: passive, moderately active, and highly active explorers. Latent transition analysis showed the moderately active explorers profile to be the most stable profile, while the passive profile was the most variable profile. Academic self-concept, motivation, test anxiety, and gender had an effect on the initial states, while motivation and test anxiety affected the transition probabilities. For both academic self-concept and motivation, students scoring higher were found to be less present in the passive or the moderately active than in the highly active profile. Furthermore, compared to students who remained in the passive profile, higher levels of motivation were associated with a higher probability to transition to the moderately active profile. Next to that, compared to students who remained in the highly active profile, higher levels of motivation were associated with a lower probability to transition to the moderately active profile. Results on anxiety were inconsistent.

**Discussion:**

Based on substantial cross-sectional as well as longitudinal data, our findings contribute to a more comprehensive understanding of the explanatory base of important differences in the study choice making process of students opting for higher education. This may ultimately lead to more timely and fitting support for students with different exploration profiles.

## 1. Introduction

The process of choosing a program in the transition to higher education is very important. The quality of this decision-making process can have an impact on choice actualization, commitment to the chosen program, and academic adjustment in higher education (Gati and Saka, 2001; Van Esbroeck et al., 2005; Skorikov, 2007; Germeijs and Verschueren, 2007a). Career exploration is a key component of study choice processes and, accordingly, to higher education adjustment and success.

In Flanders, Belgium, the higher education system is open access, with no centralized exams at the end of secondary education and no entrance exams at the start of higher education (a few exceptions such as medicine, dentistry and some art programs left alone). Unfortunately, Flemish higher education is also characterized by high levels of study delay and drop-out, implying high potential costs for individuals and society at large (OECD, 2022). In Flanders, of the students who started higher education in 2018–2019, only 30% obtained their bachelor’s degree within the predetermined study duration (Statistiek Vlaanderen, 2022a). In academic year 2019–2020, 14% of students dropped out after 1 year of higher education (Statistiek Vlaanderen, 2022b). The quality of the study choice is diverse in this context and can have an important impact on higher education success and drop-out rates (Lacante et al., 2001; Germeijs and Verschueren, 2007a). This research context is therefore interesting, in order to gain more insight into individual differences in the career exploration process of students opting for higher education in the last year of secondary education and understanding the role of antecedents. It can contribute to more evidence on the explanatory base of important differences in the career exploration process of students opting for higher education.

## 2. Theoretical framework

### 2.1. Study choice process, profiles, and transitions

The decision-making process involves a substantial amount of career exploration (Gati and Asher, 2001). Stumpf and colleagues (1983) defined career exploration as purposive behavior and cognitions that are associated with vocational development. In this process, people explore both the self and the environment to better understand their characteristics and to uncover potential career options (Porfeli and Lee, 2012). Germeijs and Verschueren (2006) identified six decisional tasks within the higher education decision-making process: orientation, self-exploration, broad exploration of the environment, in-depth exploration of the environment, decisional status, and commitment. These decision-making tasks are dynamic and flexible; there is no fixed order in which they should be tackled and tasks can be skipped or returned to as necessary (Germeijs and Verschueren, 2006; Germeijs and Verschueren, 2010). Four of these are important regarding career exploration. Orientation assesses students’ awareness of the need to decide and their motivation to make the best possible career choice. Self-exploratory behavior measures the extent to which students learn about their interests and abilities, and to what extent they discuss their attributes with significant sources of information (e.g., parents, friends, teachers). Broad exploration evaluates how much general information about higher education students research, while in-depth exploration measures the extent to which students acquire detailed information about specific career perspectives (Germeijs and Verschueren, 2006).

Most studies examining career exploration processes have used a variable-centered approach, investigating correlates of differences in variable scores for career exploration tasks. Demulder et al. (2022) complemented previous research by adopting a person-centered approach. Person-centered analysis techniques cluster or group individuals based on shared characteristics (Hickendorff et al., 2018; Woo et al., 2018) and have become increasingly common in vocational research. These analyses allow to distinguish more homogeneous groups of individuals sharing more communalities with regard to the targeted research variables in a sample, in this study, their way of engaging with the different study choice tasks for higher education. Based on students’ scores on four decisional tasks (i.e., orientation, self-, broad and in-depth exploration), Demulder et al. (2022) identified three exploration profiles using latent profile analysis: passive, moderately active and highly active explorers. However, a longitudinal design could offer supplementary insight into the variability of the exploration profiles across time. Up until now, longitudinal research addressing the development of the career decision-making process in general, and the exploration process in particular, is very scant. As one of the exceptions, Germeijs and Verschueren (2006), using a variable-centered approach, showed that, on average, students progressed a lot during the last year in secondary education and showed significant improvement in all exploration tasks. The present study aims at adding to this literature by clarifying how the career exploration process changes during the final year of secondary education of students deciding for higher education, using a person-centered approach. Particularly, latent transition analysis is useful to combine the cross-sectional measurement of categorical latent variables and the longitudinal description of change in the categories of the latent variable over time (Nylund, 2007). This allows us to further understand if and why students’ exploration profiles are variable or stable across time.

### 2.2. Antecedents

Jiang et al. (2019) present a framework of different individual and contextual antecedents that may influence career exploration in adolescence. This framework summarizes the existing evidence regarding the antecedents, outcomes, and moderators of career exploration. It shows that career exploration is powered by a mix of personal and contextual factors. Some antecedents foster exploration while others hinder it (Jiang et al., 2019). Several of these antecedents, both fostering and hindering, will be jointly examined in the present study to unravel the explanatory base of important differences in the study choice making process of students opting for higher education. In the present study, the antecedents will be jointly investigated in contrast with former research focusing more often on separate antecedents. In addition to characteristics that are fixed, we choose to also focus on malleable characteristics since the students can take action on these themselves. The antecedents that will be further focused on in the present study are the following: academic self-efficacy, academic self-concept, motivation, test anxiety, gender, educational track and socioeconomic status (SES).

Self-efficacy has been identified as a crucial factor in career exploration. Self-efficacy reflects people’s expectations and convictions about what they can achieve in given situations (Bong and Skaalvik, 2002). Research showed self-efficacy to be positively related to exploration as well as career planning, with more confident students reporting more career exploration (Creed et al., 2007; Rogers et al., 2008; Rogers and Creed, 2011). Self-efficacy also showed to be positively related to career exploration across time (Creed et al., 2007). An intervention study by Chiesa and colleagues confirmed that an increase in self-efficacy is positively associated with an increase in career exploration; improvement of self-efficacy was effective in increasing career exploration (Chiesa et al., 2016). Usually, career decision-making self-efficacy is assessed, also in the previously mentioned studies. Deng et al. (2022), however, used a measure of academic self-efficacy and investigated how this was related to different career development profiles. Their results demonstrated that the profile with the highest level of career exploration also showed a higher level of academic self-efficacy compared to profiles showing less career exploration (Deng et al., 2022).

While academic self-efficacy portrays individuals’ convictions of what they can accomplish in given situations, academic self-concept refers to individuals’ perceptions about themselves in an academic situation (Bong and Skaalvik, 2002). Academic self-concept has shown to be an important predictor for the awareness to start the career choice process for a future study (i.e., orientation) and demonstrates to be negatively associated with problems with orientation. How adolescents judge their academic abilities is related to how they think about engaging in orientation for a career in the future (van der Aar et al., 2019). Relatedly, vocational self-concept crystallization [i.e., “the degree of clarity and certainty of self-perception with respect to vocationally relevant attitudes, values, interests, needs and abilities” (Tokar et al., 2003, p. 5)] demonstrates a negative relationship with career indecision (Tokar et al., 2003; Landine, 2016), so a positive vocational self-concept can prevent career indecision and facilitate the decision-making process (Landine, 2016).

In addition, student motivation plays a part in career exploration. Motivational factors have been established as important predictors of broad and in-depth exploration, both at the between-person and within-person level (Lee et al., 2016). Research by Deng et al. (2022) showed that a career development profile highest in career exploration also showed higher levels of academic motivation compared to other profiles showing less exploration. Paixão and colleagues (2017) argued that the type of motivation might play a role. For instance, self-determined students exhibited the most positive vocational behavior. More specifically, they showed higher levels of exploration and lower levels of career indecision (Paixão and Gamboa, 2017). In line with these findings, research by Duchesne et al. (2012) demonstrated that students who actively explored showed higher levels of self-determined academic motivation. In comparison, non-self-determined students exhibited the most negative vocational behavior and showed low levels of career exploration and high levels of indecision (Duchesne et al., 2012).

Anxiety can also be linked to exploration, but results on this relationship are inconsistent. According to research, different types of anxiety may influence exploration in various ways. Career anxiety demonstrates to be positively related to environmental exploratory behavior (Vignoli et al., 2005; Germeijs et al., 2006). Career anxiety seems to decrease the processing of all non-vocational information while increasing the processing of vocational information (Vignoli et al., 2005). On the other hand, general anxiety shows to limit exploration, possibly because it is not targeted toward the academic and vocational future. General anxiety shows to incite the search for irrelevant information, which may hinder the search for academic and vocational information (Vignoli et al., 2005). However, other research by Vignoli (2015) unexpectedly showed a positive relationship between general anxiety and career exploration. Their research also showed that the fear of failing in school played a greater, positive, role than general anxiety in career exploration (Vignoli, 2015). These inconsistent results show that different types of anxiety may influence exploration differently and that even the same types of anxiety can demonstrate different relations with exploration.

Research suggests gender may also influence the decision-making process. Boys tend to make their final decision more quickly, whereas girls tend to put more effort into the decision-making process and consult others more (Gati et al., 2010). According to Germeijs and Verschueren (2006), boys often score worse than girls on the majority of decision-making tasks. Girls generally scored better on self-exploration (Seabi, 2012; Lazarides et al., 2016) or had higher levels of exploration of the environment (Gamboa et al., 2013). Another study from Germeijs and Verschueren revealed that girls scored better on the subscales orientation and broad exploration at the start of their senior year of high school. For all other tasks, there were no significant differences found at that timepoint. However, girls made more progress than boys in in-depth exploration, demonstrating higher levels of in-depth exploration at the end of secondary school (Germeijs and Verschueren, 2007b). Research from Demulder et al. (2022) associated gender with different exploration profiles and confirmed the aforementioned findings and showed that girls were more likely to be found in the highly active explorers profile compared to a moderately active or passive explorers profile. These results regarding gender could be explained by the fact that girls exhibit greater levels of engagement at school than boys do, meaning they, for instance, generally put more effort into school related tasks (Lietaert et al., 2015).

The general track of secondary education in Flanders specifically prepares students for higher education. Students who choose the technical track may already have to make a more specific and determining choice than those who opt for the general track. Therefore, students from the technical track are generally assumed to be more ready to decide than students from the general track, which seems to be why they tend to score higher on the decision-making tasks at certain moments (Germeijs and Verschueren, 2007b). However, general track students tend to make greater progress during their final year of secondary education, which possibly explains their higher scores on some decisional tasks at the end of their schooling (Germeijs and Verschueren, 2007b). Research by Demulder et al. (2022) demonstrated inconclusive results regarding the relation between educational track and different exploration profiles. Compared with technical track students, those in the general track were more likely to be moderately active than highly active explorers, but no significant difference between the passive and highly active explorers profile was found.

Finally, a higher socioeconomic status demonstrates to be positively related to the decision-making process. Students who indicated having greater economic resources, social power, and social prestige, expressed greater confidence in their capacity to carry out career decision-making tasks (Thompson and Subich, 2006; Metheny and McWhirter, 2013) and reported more certainty in their career decision (Thompson and Subich, 2006).

## 3. The present study

**Figure 1:**
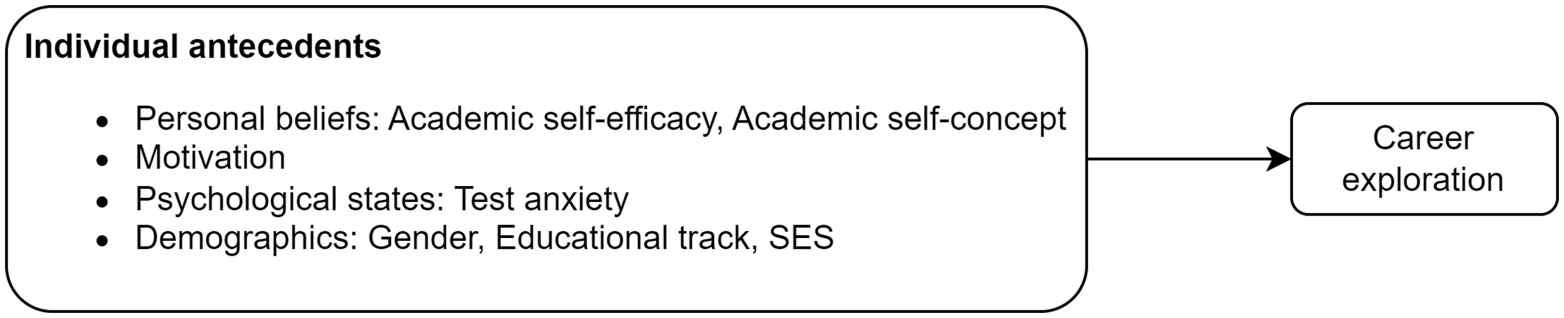
The conceptual model for this study.

The present study aimed to examine the exploration process using a person-centered approach to better understand differences in the higher education decision-making process. We first investigated the presence of exploration profiles based on four different decision-making tasks within the process (i.e., orientation, self-, broad, and in-depth exploration) cross-sectionally at two timepoints (Fall and Spring of the senior year in secondary education). Secondly, we examined in which way students transition between exploration profiles across the two timepoints. Finally, we investigated different antecedents of both profile membership and transitions between profiles. Four research questions guided this study. The first research question is as follows: “Which exploration profiles of students can be identified in Fall and Spring of the final year in secondary school?” We expected to find three exploration profiles at both timepoints: passive, moderately active and active explorers, based on previous research with a similar sample (Demulder et al., 2022). The second research question of this study was “To what extent do students transition between exploration profiles across these two timepoints?” Since research showed that students improve significantly in all exploration tasks during the last year of secondary education (Germeijs and Verschueren, 2006), we expected that if students transition between profiles they will primarily move to profiles with higher levels of exploration between Fall and Spring. The third and fourth research question both looked further into the role that different antecedents play: “Can different antecedents (i.e., academic self-efficacy, academic self-concept, motivation, test anxiety, gender, educational track, socio-economic status) explain profile membership?” and “Can these different antecedents explain who transitions between profiles?.” Based on previous research, we expected academic self-efficacy, academic self-concept, and motivation to have a positive effect on exploration. Research on the relationship between exploration and anxiety has been inconsistent, and has not yet focused much on test anxiety, but we expect it to also have a positive effect based on the research by Vignoli and colleagues (2015). So, we expected students with higher average scores for academic self-concept, academic self-efficacy, motivation, and test anxiety to have more active profiles and to be able to transition to a more active exploration profile. Since research indicates that female students score higher on both self-and environmental exploration (Germeijs and Verschueren, 2006; Germeijs and Verschueren, 2007b; Seabi, 2012; Gamboa et al., 2013; Lazarides et al., 2016) and are more likely to be in a highly active profile (Demulder et al., 2022), we expect girls to have more active profiles and to be more able to transition to a more active exploration profile. Regarding educational track, research suggests that technical track students are generally more ready to decide those from the general track, but that general track students tend to make greater progress during their final year of secondary education (Germeijs and Verschueren, 2007b). We thus expect the technical track students to have more active initial profiles but the general track students to be more able to transition to a more active exploration profile. SES has shown to be positively related to the decision-making process. Therefore we expect students with higher SES to have more active profiles and to be able to transition more easily to a more active exploration profile. Figure 1, based on the framework proposed by Jiang et al. (2019), shows the conceptual model used for this study.

## 4. Methodology

### 4.1. Participants and procedure

Data used in this study are part of data collections taking place in the Columbus project – a large-scale research initiative of the Flemish Department of Education and Training. Columbus is also the name of the exploration instrument designed to improve the career decision-making processes of students nearing the end of secondary education (Demulder et al., 2020). The instrument consists of a set of validated questionnaires and tests, which, by providing normed and personalized feedback, helps students to explore their possibilities, explains their strengths and areas for improvement, informs them about possible risks when entering higher education, and provides them with suitable remediation tips for further development.

Data from three cohorts (school years 2017–2018, 2018–2019, 2019–2020) were merged to ensure sufficient data were present for the analyses. We selected students from the general and technical tracks because they most often make the transition to higher education. Since higher education in Flanders is largely unconstrained, students can freely choose from all study programs. Furthermore, we selected those students who filled out the Shortened Study Choice Task Inventory (SSCTI) a first time in Fall and a second time in Spring and who actually started higher education in the following academic year. These two timepoints were chosen since previous research showed an increase in all exploration tasks between the first and second trimester of the senior year (Germeijs and Verschueren, 2006). For Fall the months of October and November were selected, and for Spring the months of February, March, and April. This resulted in three datasets for further analysis: a cross-sectional dataset for Fall (sample A), a cross-sectional dataset for Spring (sample B), and a longitudinal dataset (sample C). For the longitudinal dataset, an extra selection was made in that students should have completed the scales regarding the antecedents in Fall. The data were standardized and examined for outliers, defined as students who scored higher than three SD above or below the mean on the scales under investigation.

After deleting 299 students because they were outliers (*n* = 150) or answered one or both of the bogus items wrong (*n* = 153), sample A (cross-sectional Fall dataset) consisted of 9,567 students. 36.6% of these students were identified as male and 63.4% as female. Students were on average 18 years old when using the instrument. 68.5% were in the general track of secondary education and 32.5% in the technical track. Regarding higher education, no selection was made regarding the programs; all programs chosen by the students selected in the dataset were included. This resulted in a total of 160 different higher education programs being present in the final dataset for sample A. 53.4% of students were in an academic bachelor program, 45.6% in a professional bachelor, and 1% in an associate’s degree program with most students choosing a program related to economics (16.5%), healthcare (9.4%), social work (8.8%), or engineering (8.6%).

After deleting 184 students because they were outliers (*n* = 49) or answered one or both of the bogus items wrong (*n* = 138), sample B (the cross-sectional Spring dataset) consisted of 7,254 students for timepoint 2 (Spring). 40.9% of these students were identified as male and 59.1% as female. They were 18 years old when using the instrument. 59.8% were in the general track of secondary education and 40.2% were in the technical track. Regarding higher education, no selection was made regarding the programs; all programs chosen by the students selected in the dataset were included. This resulted in a total of 159 different higher education programs in the final dataset for sample B. 48.2% of students were in an academic bachelor program, 50.6% in a professional bachelor, and 1.2% in an associate’s degree program with most students choosing a program related to economics (16.6%), engineering (11.7%), healthcare (9.8%), or social work (8.9%).

After deleting 39 students because they were outliers (*n* = 6) or answered one or both of the bogus items wrong (*n* = 33), sample C (the longitudinal dataset) consisted of 672 unique cases. 31.7% of these students were identified as male and 68.3% as female. 60.1% were in the general track of secondary education and 39.9% in the technical track. Again, all programs chosen by the students selected in the dataset were included. This resulted in 102 different higher education programs being present in the data. 49.9% of students were in an academic bachelor program, 48.2% in a professional bachelor, and 1.9% in an associate’s degree program. Most students chose a program related to economics (15%), healthcare (10%), social work (9.7%), engineering (9.5%), or education (8.8%).

### 4.2. Measures

#### 4.2.1. Exploration profiles

Students completed the validated shortened and updated version of the Study Choice Task Inventory (SSCTI; Demulder et al., 2019). The Study Choice Task Inventory (SCTI) has six scales, each of which measures one of six career-decisional tasks: Orientation, Self-Exploration, Broad Exploration, In-Depth Exploration, Decisional Status, and Commitment (Germeijs and Verschueren, 2006).

In the current study, the orientation and exploration subscales were used. The subscale Orientation (*α*_*range sample* A, B, C_ = 0.79–0.82) measures students’ awareness of the need to make a study career decision, as well as their motivation for making this decision. On a 5-point scale, the students respond to 5 items. The Self-Exploratory Behavior scale (*α*_*range sample* A, B, C_ = 0.78–0.84), which consists of 8 items scored on a 4-point scale, assesses to what degree students gather information about themselves. Broad Exploration (*α*_*range sample* A, B, C_ = 0.84–0.85) assesses the extent to which students investigate general information about higher education, while In-Depth Exploration (*α*_*range sample* A, B, C_ = 0.66–0.77) measures the level of detailed information students gather about specific career options. Both scales consist of 5 items answered on a 4-point scale. Before answering the In-Depth Exploration questions, the students had to list what majors they already explored. The In-Depth Exploration scale was not required for those who had not gathered information on programs.

#### 4.2.2. Antecedents

All antecedents were measured using a combination of scales from different validated instruments. Academic self-efficacy (*α*_*range sample* A, B, C_ = 0.88) is part of the short version of the Inventory of Learning patterns of Students (ILS-SV; Donche and Van Petegem, 2008; Vermunt and Donche, 2017) and measures the students’ confidence in their capabilities and in their way of studying. Self-efficacy consists of 4 items that students answer on a 5-point scale. Academic self-concept (*α*_*range sample* A, B, C_ = 0.85) was measured by using an adjusted version of the academic subscale of the Self-Concept Scale (Wouters et al., 2011). Students answered 7 items on a 5-point scale. Motivation (*α*_*range sample* A, B, C_ = 0.71–0.75) and Anxiety (*α*_*range sample* A, B, C_ = 0.82) are two scales from the Learning and Study Strategies Inventory (LASSI; Weinstein et al., 2016). Both scales consist of 6 items that are answered on a 5-point scale. Motivation measures the students’ willingness to put up the effort required to successfully complete their academic obligations. Test anxiety assesses the degree to which students worry about school and their academic performance.

Information about students’ gender, educational track, and SES was obtained by linking the datasets to the administrative database of the Flemish Department of Education and Training, which contains information about students’ secondary education careers. Socio-economic status was operationalized as the educational level of the mother, with students with a mother without a higher secondary education degree considered as low SES.

### 4.3. Data analysis

To answer the first research question, unraveling which different exploration profiles can be identified cross-sectionally, we applied latent profile analysis (Vermunt and Magidson, 2016). A latent profile analysis (LPA) is a model-based approach in which individuals are allocated into clusters based on membership probabilities estimated directly from the model. This makes the choice of cluster criterion less arbitrary in comparison with standard cluster analysis techniques (Vermunt and Magidson, 2002; Spurk et al., 2020). With LPA, we can look into qualitatively different configurations of variables (Spurk et al., 2020).

To answer the second research question, exploring if and in which way students transition between exploration profiles at the two timepoints, latent transition analysis was used. Latent transition analysis (LTA) is a longitudinal version of latent profile analysis. It combines the cross-sectional measurement of categorical latent variables and the longitudinal description of change in the categories of the latent variable over time. LTA is a type of autoregressive model to examine time-to-time change in latent categorical variables (Nylund, 2007). LTA describes how students move between groups by providing transition probabilities that describe the probability to transition from a particular latent class to another latent class between measurement points (Sorgente et al., 2019).

For all LPA’s and LTA, the scores of the four study choice tasks orientation, self-exploration, broad exploration, and in-depth exploration from the corresponding SSCTI scales were standardized using z-scores before entering them into the analyses. We used different parameters to evaluate fit. We used the Bayesian Information Criterion (BIC) statistic to determine whether including additional classes would improve the model fit. The BIC for a solution with k classes should be lower than for a solution with k-1 classes. Entropy (E) and the classification error (CE) were used to check the classification accuracy. The closer E is to 1 and CE is to 0, the more accurate the predictions (Vermunt and Magidson, 2005). Finally, it is important to determine the number of classes not only on the fit indices, but also while considering theoretical justification, parsimony, and easiness of interpretation (Vermunt and Magidson, 2003; Jung and Wickrama, 2008). After identifying the profiles, we checked whether there were significant differences among the profiles for each of the scales included in the latent profile analysis. In the Supplementary materials two tables containing means, standard deviations, and ANOVA’s are included to examine differences between the profiles for the four exploration tasks at both timepoints. For both timepoints, the ANOVA’s showed significant profile differences. *Post hoc* tests showed significant differences between all profiles on all exploration tasks at both timepoints, with students in the highly active profile scoring higher than students in the moderately active profile, who in turn scored higher than students in the passive profile, except for in-depth exploration where no significant difference was found between the moderately active and highly active profile in Fall.

Following the LPA’s and LTA, we investigated the effect of antecedents on the initial states and the transition probabilities. The log odds ratios in Latent Gold were transformed into log odds to ease interpretation. For the effect of the antecedents on the transitions, the odds ratios describe the probability to transition to another profile compared to the probability to remain in the same profile. All analyses were performed in Latent Gold.

## 5. Results

### 5.1. Latent profile analyses

We inspected 1–6 profile solutions to identify exploration profiles in both datasets. Since the best loglikelihood value was not replicated for all profile solutions, we decided to increase the random sets and the iterations to be sure to avoid local maxima. However, after adjusting the start values multiple times, at both timepoints, not all profile solutions seemed stable. For sample A (Fall), only the profile solutions up to three showed to be stable. For sample B (Spring), the profile solutions were stable up to four. All parameters are shown in Tables 1, 2.

**Table 1:** Model fit statistics for the latent profile analysis for sample A (Fall).

Number of profiles	AIC	BIC	Δ BIC	CE	Entropy *R*^2^
1	108611.88	108669.21	/	0	1
2	102120.33	102242.15	6427.06	0.10	0.66
3	97137.22	97323.54	4918.61	0.11	0.74
4					
5					
6					

**Table 2:** Model fit statistics for the latent profile analysis for sample B (Spring).

Number of profiles	AIC	BIC	Δ BIC	CE	Entropy *R*^2^
1	82355.84	82410.96	/	0	1
2	76300.10	76417.21	5993.74	0.09	0.70
3	72057.12	72236.24	4180.97	0.10	0.77
4	70897.16	71138.29	1097.95	0.15	0.71
5					
6					

**Figure 2:**
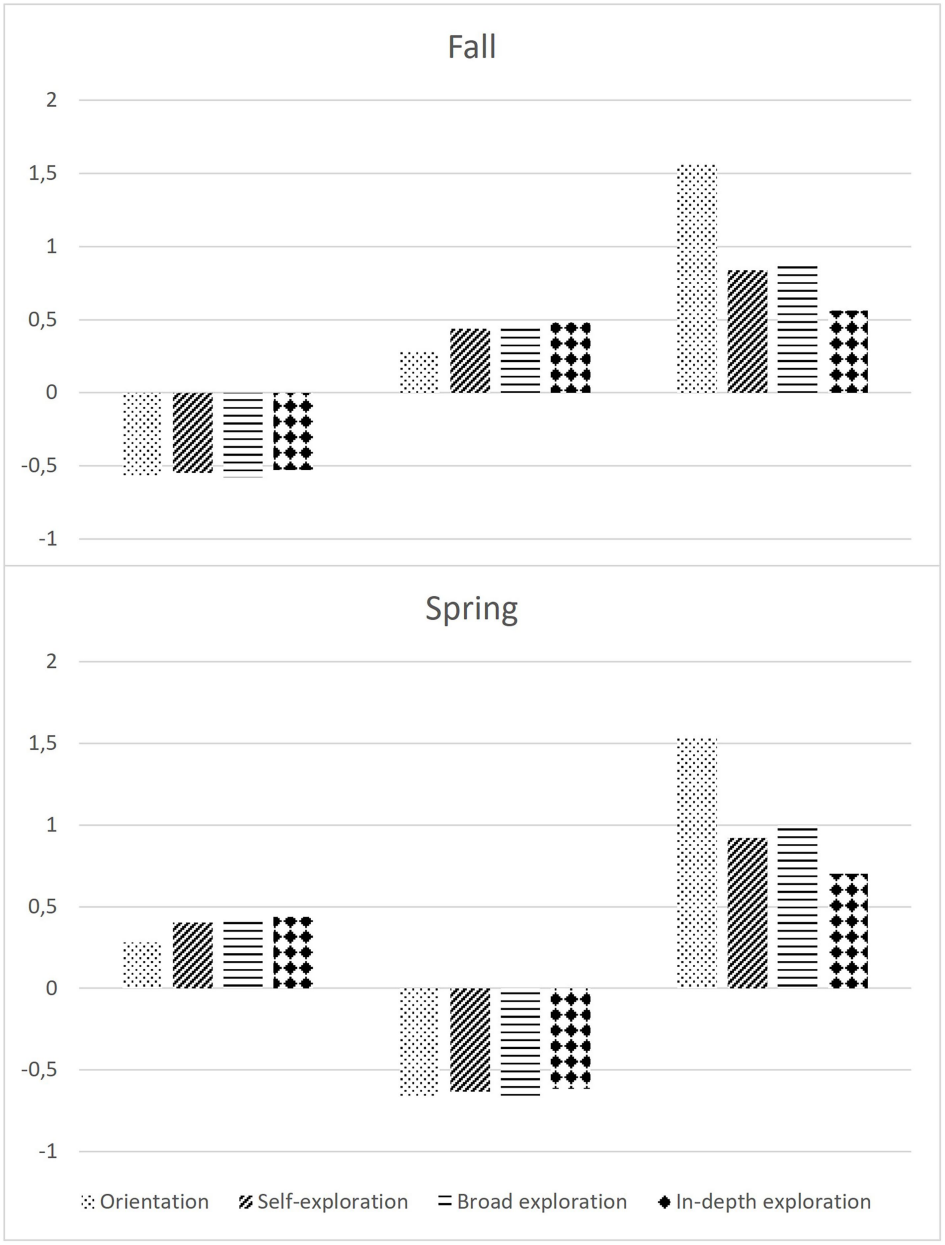
The three exploration profiles and corresponding z-scores for the four decisional tasks at both timepoints.

We used a combination of the BIC, classification error, and entropy to compare which models fit the data best. In addition, it is important to take into account the interpretability of the profiles. The statistical indices might suggest a solution that is not a useful model for capturing the population’s heterogeneity. So, when choosing the number of profiles, it is important to take into account both statistical and practical considerations (Nylund, 2007). Hence, with theory, parsimony, interpretability, and stability in mind, and to ensure sufficient differentiation between the profiles, at both timepoints, we chose the three-profile solution over other solutions indicating more profiles. The final three-profile solutions are presented in Figure 2. The profiles are depicted using z-scores. The profile with students scoring relatively low on the four decisional tasks was labeled as the ‘passive explorers’. This profile was most present in our data for sample A (48%) and the second largest for sample B (44%). Students scoring moderately on all four decisional tasks were named the ‘moderately active explorers.’ This was the second largest profile in sample A (41%) and the largest profile in sample B (45%). At both timepoints, the ‘highly active explorers’ were least present in the data (respectively 10 and 11%) and scored relatively high on all four decisional tasks.

### 5.2. Latent transition analysis

For the LTA, we first checked the longitudinal sample cross-sectionally using the same procedure and parameters as in the earlier stage to determine the number of profiles, or states, as they are called in LTA. As in samples A and B, the parameters showed the three-profile solution to be most suitable at both timepoints in sample C. At both timepoints, the ‘moderately active explorers’ were the largest profile, representing, respectively, 46% and 49% of students. The ‘passive explorers profile’ made up, respectively, 43% and 35% of students. At both timepoints, the ‘highly active explorers’ were the smallest group within the dataset (respectively 11% and 16%). Based on these cross-sectional results, we proceeded with the LTA of the longitudinal data with three latent statuses. The pattern of transition between groups is presented in Table 3.

**Table 3:** Latent transition probabilities between profiles across Fall and Spring.

Fall	Spring		
	Moderately active	Passive	Highly active
Moderately active	0.779	0.001	0.220
Passive	0.440	0.519	0.041
Highly active	0.418	0.005	0.577

The ‘moderately active explorers’ proved to be the most stable profile, with 78% staying in the same profile between Fall and Spring. Of the students who were in the ‘moderately active explorers’ profile in Fall, 22% transitioned to the ‘highly active’ profile in Spring. For the ‘passive’ profile, 52% remained in the same profile across time. 44% of them transitioned to the ‘moderately active’ profile, while 4% transitioned to the ‘highly active’ profile. Of the ‘highly active explorers,’ 58% stayed in the same profile. However, 42% of them transitioned to the ‘moderately active’ profile.

### 5.3. Antecedents

Following the LTA, we tried to further unravel the effect of different antecedents. First, we checked the association of antecedents with the initial states. The results showed that test anxiety [Wald *χ^2^* (2) = 17.94, *p* < 0.001], academic self-concept [Wald *χ^2^* (2) = 20.58, *p* < 0.001], motivation [Wald *χ^2^* (2) = 21.13, *p* < 0.001] and gender [Wald *χ^2^* (2) = 6.34, *p* < 0.05] had a significant relation with profile membership. Students scoring higher on test anxiety were less likely to be included in the ‘passive’ profile than in the ‘highly active’ profile (OR = 0.44, *p* < 0.001). For academic self-concept students scoring higher were found to be less present in the ‘passive’ (OR = 0.16, *p* < 0.001) or the ‘moderately active’ profile (OR = 0.35, *p* < 0.05) than in the ‘highly active’ profile. Also for motivation students scoring higher were less likely to be included in the ‘passive’ (OR = 0.21, *p* < 0.001) or the ‘moderately active’ profile (OR = 0.46, *p* < 0.05) than in the ‘highly active’ profile. For gender, only the overall effect was significant. For academic self-efficacy, educational track, and SES no significant associations with the initial states were found.

In a final step, we measured the effect of antecedents on the transitions between profiles. Males were the reference group for gender, technical track was the reference group for educational track, and having a mother without a higher secondary education degree was the reference group for SES. The results showed that both motivation [Wald *χ^2^* (6) = 25.30, *p* < 0.001] and test anxiety [Wald *χ^2^* (6) = 13.14, *p* < 0.05] had an overall significant effect on transition probabilities between profiles. Compared to students who remained in the ‘passive’ profile, higher levels of motivation were associated with a higher probability to transition from the ‘passive’ to the ‘moderately active’ profile (OR = 4.74, *p* < 0.001). Further, compared to students who remained in the ‘highly active’ profile, higher levels of motivation were associated with a lower probability to transition from the ‘highly active’ to the ‘moderately active’ profile (OR = 0.09, *p* < 0.05). For motivation, no significant effect was found for the transitions from and to the other profiles. For test anxiety, compared to students who remained in the ‘moderately active’ profile, higher levels of test anxiety were associated with a higher probability to transition from the ‘moderately active’ to the ‘highly active’ profile (OR = 2.02, *p* < 0.05). In addition, compared to students who remained in the ‘passive’ profile, higher levels of test anxiety were associated with a lower probability to transition from the ‘passive’ to the ‘highly active’ profile (OR = 0.07, *p* < 0.05). No significant effect was found for the transitions from and to the other profiles. For gender there was no overall significant effect, but compared to students who remained in the ‘passive’ profile, girls had a higher probability than boys to transition from the ‘passive’ to the ‘moderately active’ profile (OR = 2.21, *p* < 0.05). For academic self-concept, academic self-efficacy, educational track, and SES no significant effects on the transition probabilities were found.

## 6. Discussion

The present study aimed to further unravel individual differences present within the decision-making process of last year students in secondary education making a study choice for higher education. To that end, we examined students’ exploration profiles during Fall and Spring of the final year before they entered higher education, both cross-sectionally and longitudinally, and sought to examine if different antecedents (i.e., academic self-efficacy, academic self-concept, motivation, test anxiety, gender, educational track, socio-economic status) could explain the initial states and transitions between profiles.

Our first objective was to identify different exploration profiles cross-sectionally at two timepoints, Fall and Spring of the last year before transitioning to higher education. We chose to adopt a person-centered approach to deepen the understanding of how different exploration tasks jointly combine into meaningful profiles. We chose a LPA over standard cluster analysis techniques because the model-based approach makes the choice of the cluster criterion less arbitrary (Vermunt and Magidson, 2002). The LPA on four decision-making tasks revealed the presence of three exploration profiles at both timepoints. The passive explorer profile, which we found to be relatively low to unengaged in terms of orientation and exploration regarding their study choice, consisted of 48% of students in the Fall and 44% of students in Spring. Other students, more specifically 41% of students in Fall and 45% of students in Spring, were slightly more aware of the need to decide and were in the process of exploring both themselves as well as the higher education environment. Accordingly, they were labeled the moderately active explorers. At both timepoints, a minority of students (respectively 10 and 11%) were part of the profile of students that already made work of orientation as well as all three exploration tasks (i.e., self-exploration, broad and in-depth exploration of the study environment). They were labeled the highly active explorers. These findings attest to the robustness of the three profile solution across timepoints and samples. The same three distinct profiles as in previous research with a similar, but only one, sample and at only one timepoint arise (Demulder et al., 2022). Given the variety of exploration profiles that were revealed cross-sectionally as well as longitudinally, our findings underline the various ways that students engage with the transition from secondary to higher education and show the possibility of detecting at-risk students in terms of the exploration activities they show or do not show across time. Student counselors should embrace this diversity and not use the same guidance for all students. Tailored counseling could be developed to more effectively match students’ exploration profiles. For example, the passive explorers, who often might have yet to fully start the decision-making process, could be more activated to put more effort into the exploration process and given guidance on how to start gaining information about the self and career alternatives (see also Demulder et al., 2022). Also, students who remain in the passive profile over time may need more intense guidance and counseling than students whose lack of exploration is only temporary.

Our second objective was to investigate the within-person transition probabilities between profiles across the two timepoints. Our results showed that the passive profile was the most variable profile, with just 52% of students staying in this profile. So, almost half of them are able to make a ‘positive’ transition, moving mostly to the moderately active profile (44%) and rarely to the highly active profile (4%). In addition, 22% of students are able to transition from the moderately active to the highly active profile, even though it is the most stable profile with 78% of students staying in this profile between Fall and Spring. Most students being able to make a positive transition is in line with previous variable-centered research showing general increases during the last year in the degree of exploration (Germeijs and Verschueren, 2006). Students seem to be making progress in both orientation and exploration during their last year of secondary education, which could be why they are most often able to move out of the passive profile to the two other profiles demonstrating more exploration. Rather unexpectedly, students from the highly active profile also transitioned quite commonly to the moderately active profile (42%). However, the highly active profile was the smallest in the data so in absolute numbers it concerns only a small fraction of students. It could be that after a period of high exploration behavior, a subsequent period with less exploration occurs since the students may have already gone through the most important phase of their decision-making process. Transitions from the highly active or the moderately active to the passive profile were almost absent. These results show that moving to the moderately active profile is the most common while transitioning to the passive profile is the least common. Transitioning from the passive exploration profile is more common than transitioning from the other two exploration profiles. However, 52% of students remained in the passive profile between Fall and Spring. A possible explanation for this could be that a number of “stayers” do not see the need to change and thus do not undertake any action. It could also be that they started their exploration process between Fall and Spring but gave up in the meantime. The research on transitions between profiles adds to the understanding of the timing of interventions to help students in their decision-making process. Early tracking of students having a passive exploration profile in Fall may help to take targeted actions to prevent them from remaining in this profile over time. Effective interventions might have a beneficial impact on this group of students, of whom more than half seem to remain more passive in their study choice making process.

Our final objective was to further unravel the association with different antecedents. The current study was able to test multiple antecedents from the framework by Jiang et al. (2019) to unravel the explanatory base of differences in the career exploration process of students opting for higher education. In addition to characteristics that are fixed, we chose to also focus on malleable characteristics since the students can take action on these themselves and they can be set in motion by interventions and counseling. First, we checked the associations of antecedents with the initial states. Subsequently, we examined the effect of different antecedents on the latent transitions between profiles. Results only partially confirmed our hypotheses. Academic self-concept was only associated with the initial states. Students scoring higher were less likely to be in the passive or moderately active profile than in the highly active profile for the initial states. Variable-centered research findings showed how academic self-concept is important for the awareness to start the orientation process and this relationship seems also important to understand differences in students’ exploration profiles (van der Aar et al., 2019). Since students with a lower self-concept have less clear perceptions about themselves, they could experience problems with the integration of self- and environmental information. It may be more difficult for them to match new incoming information with their existing knowledge about themselves since this knowledge is still unclear. A clear and certain self-concept could thus facilitate the exploration process.

Motivation proved to be associated with the initial states as well as with the transition probabilities. Students scoring higher were less likely to be in the passive or moderately active profile compared to the highly active profile for the initial states. Moreover, high levels of motivation were associated with a higher probability to remain in the highly active profile compared to transitioning from the highly active to the moderately active profile and associated with a higher probability to transition from the passive to the moderately active profile compared to remaining in the passive profile. Variable-centered research confirmed the importance of and positive relation between motivation and exploration (Duchesne et al., 2012; Paixão and Gamboa, 2017; Deng et al., 2022). The current study demonstrates that motivation is important to understand differences in students’ exploration profiles, hence explaining why students scoring higher on motivation can more often be found in the highly active profile compared to the moderately active or passive profile. Moreover, motivation seems to protect students in the highly active profile from moving to the moderately active profile, and it supports students in transitioning from the passive to the moderately active profile. Since students with higher motivation are more willing to invest effort in academic requirements, these motivations may also play a role in students’ engagement in career planning activities. Students willing to invest more effort in academic tasks may also be more willing to invest more effort in dealing with the different tasks in the decision-making process.

Test anxiety was also associated with both the initial states and the transition probabilities. Students scoring higher were less likely to be included in the passive profile than in the highly active profile, so highly active students do not seem to show well-adjusted behavior regarding all antecedents. As mentioned above, results on the relationship between anxiety and exploration are inconsistent, depending on the type of anxiety that is measured. Both career (Vignoli et al., 2005; Germeijs et al., 2006) and test (Vignoli, 2015) anxiety proved to be positively related to exploration, while general anxiety shows confusing results, with some studies showing a negative relation (Vignoli et al., 2005) and others a positive relationship with exploration (Vignoli, 2015). The measure of anxiety used in the current study assessed the degree to which students worry about school and their academic performance. Highly active explorers show higher test anxiety than their passive counterparts, which could explain why they also show more exploration. Regarding the transition probabilities, high levels of test anxiety were associated with a higher probability to transition to the highly active profile compared to staying in the moderately active profile and associated with a higher probability to stay in the passive profile compared to transitioning to the highly active profile. On the one hand, our results show that among students that do explore, over time higher test anxiety may lead to increased engagement in exploration. On the other hand, test anxiety may hinder passive explorers to become more highly engaged in exploration. So, depending on the individual, test anxiety may have different consequences, either hindering growth in exploration or fostering it. Being aware of the differential impact of anxiety for different individuals may be useful for guiding them in the decision-making process. It would be interesting for future research to investigate if the group of highly active explorers with high test anxiety encounter more difficulties deciding on and committing to a study choice. It should be noted, however, that anxiety only influenced the transition from the moderately active to the highly active profile and from the passive to the highly active profile. There was no significant influence on the transition probabilities to and from the other profiles.

Finally, girls had a higher probability than boys to transition to the moderately active profile compared to staying in the passive profile. Previous variable-centered research on gender has shown that girls report higher levels of exploration of the environment (Gamboa et al., 2013) and self-exploration (Seabi, 2012; Lazarides et al., 2016) and that girls make more progress than boys in in-depth exploration, showing higher levels at the end of secondary school (Germeijs and Verschueren, 2007b).

For the other antecedents (i.e., academic self-efficacy, educational track, and SES) no significant associations with the initial states or the transition probabilities were found. A possible explanation for this could be that the variance is already explained by other variables in the model. For self-efficacy, another explanation could be the form of self-efficacy used in the present study. Results could have been different when for instance career decision-making self-efficacy was used. Most research on self-efficacy used career decision-making self-efficacy (Creed et al., 2007; Rogers et al., 2008; Rogers and Creed, 2011), while the current study used a measure of academic self-efficacy measuring students’ confidence in their capabilities and in their way of studying.

Since this study showed that academic self-concept, motivation, and test anxiety are associated with the initial exploration profile and that motivation and test anxiety can have an effect on the transition probabilities, interventions and counseling taking into account these antecedents could be beneficial to help students to better cope with the challenges of the decision-making process. For instance, students with a more positive academic self-concept are more often highly active explorers. Working on a more positive self-concept could indirectly also improve their exploration process or profile. In addition, as motivation shows to be positively related to transitioning from the passive to the moderately active profile, interventions aimed at increasing motivation could be beneficial. Research by Chiesa et al. (2016) has already shown that improving self-efficacy through an intervention can also increase career exploration. In the current study, test anxiety was associated with both the initial exploration profiles and the transition probabilities but showed confounding results. It could thus be useful for counselors to be aware of the different types of anxiety to help students manage their anxiety and assist them in the decision-making process.

### 6.1. Limitations and directions for future research

The current study has some limitations which, if addressed, might also offer suggestions for future research. First, the LPA’s and LTA were based on self-report measures, which may have allowed for bias as students may have answered questions in a socially desirable way. However, based on substantial samples investigated in this study, both in the LPA’s and LTA quite a diversity of exploration profiles and transitions between profiles were found, not only indicating so-called positive exploration profiles. A second limitation of the current study is the fact that only two timepoints were taken into account. Taking into account three or more timepoints would be interesting to better understand the linear trend of the exploration process. For instance, an extra timepoint at the very end of the academic year, before leaving secondary education, could possibly bring useful insights. It would also be interesting to measure the antecedents at multiple points in time so changes in antecedents over time can be associated with transitions between profiles. Third, this study was able to test several, but not all, antecedents from the framework by Jiang et al. (2019). Future research could build on the present study by further examining the other antecedents from the framework in a person-centered way. Their review study has shown how different antecedents are related to career exploration. Less is known, however, about how these relations occur when using a person-centered approach. For instance, the role of career interests, or especially different contextual antecedents such as school or parental support in study choice guidance could be further investigated as predictors of profile membership or transition probabilities.

## Data availability statement

The datasets presented in this article are not readily available given the confidential nature of the data. Requests to access the datasets should be directed to lien.demulder@kuleuven.be.

## Ethics statement

Ethical review and approval was not required for this study on human participants in accordance with the local legislation and institutional requirements. The datasets on which this study is based are part of a larger project, Columbus, commissioned by the Flemish Ministry of Education and Training. Written informed consent was obtained from all participants. When registering, students agree to the terms and conditions formulated in collaboration with the Data Protection Officer of the Ministry of Education and Training and in accordance with the General Data Protection Regulation (GDPR). The Ministry strictly follows the principles of the GDPR when processing personal data. The sharing of personal data requires a protocol. The Columbus protocol (January 27, 2021) can be found at https://data-onderwijs.vlaanderen.be/documenten/bestand.ashx?id=13051.

## Author contributions

LD performed the study and wrote the first draft of the manuscript. All authors contributed to the study concept and design, discussed the data analyses, commented on previous versions of the manuscript, and read and approved the final manuscript.

## Conflict of interest

The authors declare that the research was conducted in the absence of any commercial or financial relationships that could be construed as a potential conflict of interest.

## Publisher’s note

All claims expressed in this article are solely those of the authors and do not necessarily represent those of their affiliated organizations, or those of the publisher, the editors and the reviewers. Any product that may be evaluated in this article, or claim that may be made by its manufacturer, is not guaranteed or endorsed by the publisher.
