# Supplementary material for: Understanding transitions in exploration profiles of students opting for higher education
Source: Front Psychol. 2023 Feb 9;14:1085718. doi: 10.3389/fpsyg.2023.1085718 (PMC9948654; doi:10.3389/fpsyg.2023.1085718)
Supplement: Supplementary file 1 [file Table_1.DOCX]

**Supplementary Table 1**

*Means, Standard Deviations, and One-Way Analyses of Variance in the four exploration tasks for the three profiles in Fall*

|  | Passive | | Moderately  active | | Highly  active | | *F*(2, 9564) | η² |
| --- | --- | --- | --- | --- | --- | --- | --- | --- |
|  | *M* | *SD* | *M* | *SD* | *M* | *SD* |  |  |
| Orientation | 3.83 | .46 | 4.33 | .35 | 5.00 | .00 | 4255.33* | .47 |
| Self-exploration | 2.40 | .35 | 2.91 | .40 | 3.05 | .49 | 2371.44* | .33 |
| Broad exploration | 1.92 | .44 | 2.66 | .55 | 2.88 | .69 | 2866.06* | .38 |
| In-depth exploration | 1.58 | .34 | 2.19 | .53 | 2.19 | .65 | 2058.95* | .30 |

* *p* < .001.
