# Supplementary material for: Understanding transitions in exploration profiles of students opting for higher education
Source: Front Psychol. 2023 Feb 9;14:1085718. doi: 10.3389/fpsyg.2023.1085718 (PMC9948654; doi:10.3389/fpsyg.2023.1085718)
Supplement: Supplementary file 2 [file Table_2.docx]

**Supplementary Table 2**

*Means, Standard Deviations, and One-Way Analyses of Variance in the four exploration tasks for the three profiles in Spring*

|  | Passive | | Moderately  active | | Highly  active | | *F*(2, 7251) | η² |
| --- | --- | --- | --- | --- | --- | --- | --- | --- |
|  | *M* | *SD* | *M* | *SD* | *M* | *SD* |  |  |
| Orientation | 3.66 | .51 | 4.25 | .37 | 5.00 | .00 | 3867.23* | .52 |
| Self-exploration | 2.40 | .35 | 2.95 | .42 | 3.18 | .50 | 2142.82* | .37 |
| Broad exploration | 2.19 | .43 | 2.93 | .48 | 3.25 | .57 | 2767.48* | .43 |
| In-depth exploration | 1.83 | .41 | 2.56 | .56 | 2.68 | .71 | 1886.53* | .34 |

* *p* < .001.
